# Our Journey Westward

**Published:** 1895-10

**Authors:** 


### OUR JOURNEY WESTWARD.

Through the usual forethought of Dr. Hoskins the Eastern delegation was provided at Philadelphia with a special Pullman sleeper to Chicago, where we arrived after a very pleasant and uneventful though very warm trip, on Sunday morning the 8th. Waiting at the depot we found a number of coaches in charge of Dr. Schwarzkopf, representing Dr. McKillip. We were escorted directly to the beautiful residence of Dr. McKillip, where we were met by the doctor and his estimable wife, who extended to us a hearty welcome and invited us to dinner, which was ready and waiting. After having satisfied our appetites we adjourned to the reception-room, where we met Drs. Merrilat, Kreiger, and members of Dr. McKillip's family. After being very pleasantly entertained by the host for a while we were invited to inspect the McKillip College, which impressed us as an exceptionally well-equipped institution.

After spending an enjoyable hour at the college, where the party was increased by the arrival of Drs. Lyman and Osgood, we enjoyed a continuance of Dr. McKillip's hospitality, first in a delightful drive around the city and through the parks and later in a grand dinner at the Palmer House. Here we were joined by Dr. Salmon, and the whole party made merry till the time of train departure.

On our arrival at Des Moines the morning of the 10th we found that the State Agricultural Fair was being held, and that nearly every room in the hotel was either engaged or occupied. Doubling up was therefore necessary, and as many as five of the members were in one room.

A little later we learned that the Iowa State Veterinary Medical Association was also in session and was then occupying one of the parlors of the hotel, thirty or forty members being present.

The day was spent in visiting the State Fair, and tired out with the long trip, all but the President and Secretary retired early. The two officers, however, were engaged till the small hours of the next day getting their books and business in shape for the meeting.

The next morning at about 9 o'clock the *Comitia Minora* met and transacted all the business presented, and shortly after 10 o'clock the Secretary called the roll, and the Thirty-second Annual Meeting of the United States Veterinary Medical Association was declared in session.

The Governor being unavoidably absent, Hon. W. I. Richards, the Governor's Secretary, welcomed in a very pleasing manner the members of the Association to the city of Des Moines, extended to them the privileges of the State buildings, and made profuse apologies for the Governor's absence. This was followed by Dr. Hoskins' address, which was a characteristically strong, thoughtful, inspiriting appeal for continued advance along professional lines, and for general united good work.

The routine work was then taken up and disposed of with surprising alacrity, a result due to the untiring energy and efforts of both the President and Secretary.

The next day was occupied in the reading of the report of the Committee on Diseases, which was very lengthy and somewhat tiresome. Such a report should be edited by the committee before it is read, and only the essential portions allowed to take up the time of the meeting.

The following papers were read, and the discussion was general and very interesting: "Tuberculosis," by Drs. Trumbower and Niles; "The Horse as a Producer of Antitoxine," by Dr. Schwarzkopf; "Accidents Incident to the Casting and Confining of Animals for Surgical Operations," by Dr. Tait Butler; "Roaring and Its Operative Treatment," by Dr. S. J. J. Harger; "An Important Method of Plantar Neurectomy,"[1] by Dr. H. M. McKillip; "The Therapeutics of Colic,"[2] introducing an original and important classification of colics, by Dr. W. L. Williams; "Hypodermatic Cathartics," by Dr. M. H. Reynolds; "Anasthesia in Horses," by Dr. J. C. Meyer, Jr. (Dr. Meyer demonstrated the method of chloroforming horses employed by him.)

In the evening of the second day the Iowa State Veterinary Medical Association extended an invitation to the members of the United States Veterinary Medical Association to attend the theatre in a body and witness Roland Reed in the play of the "Politician." During the several acts the members of the Association were impersonated, much to the amusement of the members in the audience.

The customary annual banquet was held at Evan's Café, and about thirty of the members sat down and enjoyed an excellent "prohibition" menu. Cider, tea, coffee, and milk constituted all that there was to appease the thirst, but the forethought of one very thirsty member sent him just a few minutes before the legal time-limit was placed upon the sale of liquor, across the way to the hotel, whence a generous supply of the necessary "thirst-quellers" followed him on his return to the table.

The guests were Private Secretary Richards, Congressman J. H. Hull, and Mr. Harold Sorby.

The trip home was fraught with all sorts of incidents. From miscalculations as to the departure of the trains and freight wrecks our arrival home was delayed more than a day.

[1] See page 638. [2] See page 613.

